# Role of multi-strain probiotics in preventing severity and frequency of recurrent respiratory tract infections in children

**DOI:** 10.1186/s12887-023-04338-x

**Published:** 2023-10-11

**Authors:** Imran Khan Laghari, Tahira Nawaz, Sultan Mustafa, Abid Ali Jamali, Sanober Fatima

**Affiliations:** 1 Abbassi Shaheed Hospital, Karachi, Pakistan; 2 National Institute of Child Health, Karachi, Pakistan

**Keywords:** Infections, Respiratory tract, Probiotics, Frequency, Pakistan

## Abstract

**Background:**

Respiratory tract infections are among the most common infections in the pediatric population throughout the globe. Globally around 20% of all deaths in children below 5 years of age are secondary to acute respiratory infections, mostly pneumonia. Probiotics are live microorganisms that when administered in adequate amounts confer a health benefit on the host. Their mechanism in preventing respiratory tract infections is not known but it is thought that probiotics act by modulating the immune system. This study was conducted to find out whether using probiotics is effective in decreasing the severity and frequency of recurrent respiratory tract infections or not.

**Methods:**

A Quasi-experimental study was conducted at the Pediatric Medicine Department of Abbassi Shaheed Hospital Karachi during 2021–2022. The study was approved by the institutional ethical review committee i.e. advanced studies and research board (ASRB). The sampling technique was non-probability consecutive sampling and the sample size was 70 patients with recurrent respiratory tract infections, aged six months to 12 years of age. All enrolled children were given probiotics containing Bifidobacterium, Lactobacillus Acidophilus for two weeks. Data were analyzed by using SPSS version 22. A p-value of < 0.05 was considered statistically significant.

**Results:**

Out of 70 children with recurrent respiratory tract infections, 39 (55.71%) were male and 31 (44.29%) female. Around 75% of the children were below five years of age. The most common presenting complaint was fever (72.86%), followed by cough (68.57%), wheezes (45.71%) and nasal discharge/sneezing (32.86%), respectively. The most common RRTI was infectious rhinitis (30% of the cases), otitis media (24%) and pharyngitis/tonsillitis (21%). After giving probiotics for two weeks most significant decrease was found in recurrent infectious rhinitis (p-value 0.02), recurrent otitis media (p-value 0.03) and recurrent bronchiolitis (p-value 0.05) over the next six months.

**Conclusion:**

The results of our study indicate that the administration of probiotics reduces recurrent respiratory tract infections among children. This six months trial has demonstrated that there was a significant decline in respiratory symptoms among study participants. This study also observed a significant decrease in respiratory diseases during the follow-up.

## Background

Respiratory tract infections (RTIs) are among the most common infections in the pediatric population throughout the globe. In developing countries like Pakistan, RTIs (especially pneumonia) are among the leading cause of mortality in children below five years of age [1]. Globally around 20% of all deaths in children below five years of age are secondary to acute respiratory infections, mostly pneumonia [2]. Pneumonia is the leading cause of death in children with worldwide deaths reported up to 740,180 children under the age of 5 in 2019. Most of these deaths were reported in developing countries from South Asia and sub-Saharan Africa [3]. As per WHO data, pneumonia cause 11.5% of deaths in children below five years of age in Pakistan and is the leading cause of death after the neonatal age period during which prematurity and birth asphyxia are the leading causes [4].

Recurrent respiratory tract infections (RRTIs) are common in children, especially in infants and young children, primarily due to their naïve immune system that is not sensitized to environmental pathogens. Studies show that 25% of infants and 6% of children below six years experience RRTIs [5, 6]. RTIs are also one of the leading causes of inadvertent antibiotic administration, thereby increasing their resistance [7]. Due to the high burden of RRTIs, studies have been done on alternative strategies for their prevention in children. Some studies have shown that probiotics and vitamin C supplementation decrease the duration and frequency of RRTIs [8, 9], while others found no beneficial effect of both these strategies [10, 11].

Probiotics are live microorganisms, when administered in adequate amount confer a health benefit on the host [10]. Although gut probiotics are thought to adhere to the mucosal lining, thereby preventing infective organisms from adherence to mucosa and causing disease, their mechanism in preventing respiratory tract infections is not known. It is supposed that probiotics act by modulating the immune system [12], but this hypothesis was also nullified by Garaiova et al. who found no significant change in cytokines [13].

Respiratory tract infections are common in Pakistan and other developing countries, primarily due to malnutrition, poor vaccination coverage, poor sanitation, large joint families and many other issues. Unfortunately, no proper strategies are developed and no research is done in this part of the world on the factors to decrease RRTIs. Clinical research using probiotic formulations has shown that it improves GI and immunological functioning in young infants and children. Unfortunately, no significant data is available (especially from low-middle-income countries) on whether these formulations are effective in preventing RRTIs and decreasing their severity or not. Therefore, we conducted this study on the local population to find out whether this simple strategy of using probiotics is effective in decreasing the severity and frequency of RRTIs or not. If found effective this may be incorporated into the national health system and policymakers be notified to start mass usage of probiotics for high-risk children.

## Methods

### Operational definition

#### Respiratory tract infections

infection of upper or lower airways i.e. infectious rhinitis, otitis media, pharyngotonsillitis, bronchiolitis, pneumonia.

#### Probiotics

definitions were taken as per WHO i.e. live microorganisms that when administered in adequate amounts confer a health benefit on the host [14]. For this study, we will give multi-strain probiotics containing bifidobacterium and lactobacilli.

#### Recurrent respiratory tract infections

Patients were labelled as having recurrent respiratory tract infections if he/she has a history of at least one of the following,

- **Infectious rhinitis**: more than five episodes per year [15].
- **Otitis media**: three or more episodes within 6 months or four or more episodes within 12 months [15].
- **Recurrent pharyngitis/tonsillitis**: more than three episodes within 12 months [15].
- **Recurrent pneumonia**: two or more episodes of radiologically documented pneumonia in a single year or three or more episodes ever, with a normal chest x-ray between episodes [16].
- **Recurrent bronchiolitis**: two or more episodes within the last 6 months or ≥ 3 episodes in a year [16].

#### Severity of recurrent respiratory tract infections

severity of recurrent respiratory tract infections was determined based on the need for hospitalizations, IV antibiotics, high-flow oxygen, and assisted ventilation.

#### Frequency of recurrent respiratory tract infections

frequency was determined based on episodes of respiratory tract infections.

### Study design and study settings

It was a quasi-experimental study, conducted at the Department of Pediatrics at Abbassi Shaheed Hospital Karachi, during 2021–2022.

### Sample size and sampling

By using openepi.com online sample size calculator version 3 and taking confidence level as 95%, power as 80%, and risk/prevalence difference of 16 [13], the calculated sample size was 70. The sampling technique was non-probability consecutive sampling.

### Inclusion criteria and exclusion criteria

Children of both sex and aged 6 months to 12 years with a history of recurrent respiratory tract infections (as per definition) were included in the study. Patients with underlying chronic respiratory tract problems (like cystic fibrosis or ciliary dyskinesia), immunodeficiency and those who are receiving probiotics due to any other underlying pathology (like inflammatory bowel disease) were excluded. Children who were given influenza vaccine were also excluded as were the children whose parents refused to participate in the study.

### Ethical approval and consent to participate

This study was approved by the ethical review committee that is advanced studies and research board (ASRB) of the University of Karachi and was allotted the reference number ASRB/No./07104/MS/MD. All patients fulfilling the inclusion criteria were selected. Informed consent for the children was obtained from all the parents or their legal guardians involved in the study. Wherever possible informed assent was also taken from the child included in the study (like children above 7 years of age). Possible benefits and any risks from this study were explained to parents/guardians. All methods were carried out per relevant guidelines and regulations in the declaration of Helsinki. Parents were given the right to withdraw at any time. Confidentiality and expertise were ensured to parents/guardians.

### Data collection

Patients with recurrent respiratory tract infections as diagnosed by the trained paediatrician were enrolled in the study. All enrolled children were given probiotics containing Bifidobacterium, Lactobacillus Acidophilus, (like Ecotec (SEARLE, Pakistan) containing > 2 billion cfu freeze dried mixture of four different microbial strains: Lactobacillus acidophilus, LA-5, Bifidobacterium, BB-12, Streptococcus thermophiles, STY-31, and Lactobacillus delbrueckii subsp Bulgaricus, LBY-27). One sachet was given orally once a day for 2 weeks. Patients were observed for 6 months for the recurrence of respiratory tract infection. Follow-up was taken by a trained team member either on the phone call or designated follow-up at the decided time that was after 6 months of enrollment and given probiotics. Data were entered in a predesigned proforma for both groups.

Data was collected from the parents/guardian of the patients through a paper-printed proforma especially designed for this study. Data was collected by a team member, who was trained prior to this sample collection. RRTIs were labelled only when diagnosed by a qualified paediatrician. After filling out the proforma, the researcher evaluated the results of the study to determine the severity and frequency RRTIs.

### Data analysis

Data were analyzed by using SPSS version 22. Study outcomes were performed using tabulations and graphs for categorical variables. A p-value of < 0.05 is considered statistically significant. Categorical variables are expressed in numbers and percentages. Continuous variables presented as mean (± standard deviation) with the range and the chi-square tests used to evaluate categorical variables used in the study.

## Results

Out of 70 children, 39 (i.e. 55.71%) were male and 31 (i.e. 44.29%) female with male to female ratio of 1.2:1. Most of the children were below 12 months of age (i.e. 41.43%) and overall around 75% of the children were below 5 years of the age, Table 1.

The most common presenting complaints among the patients with recurrent respiratory tract infections were fever, which was present in 72.86% of the patients, followed by cough (68.57%), wheezes (45.71%) and nasal discharge/sneezing (32.86%), respectively. Other common presenting complaints were earache/ear discharge (27.14%), sore throat (25.71%), respiratory distress (12.86%) and headache (8.57%). Table 2.

**Fig. 1:**
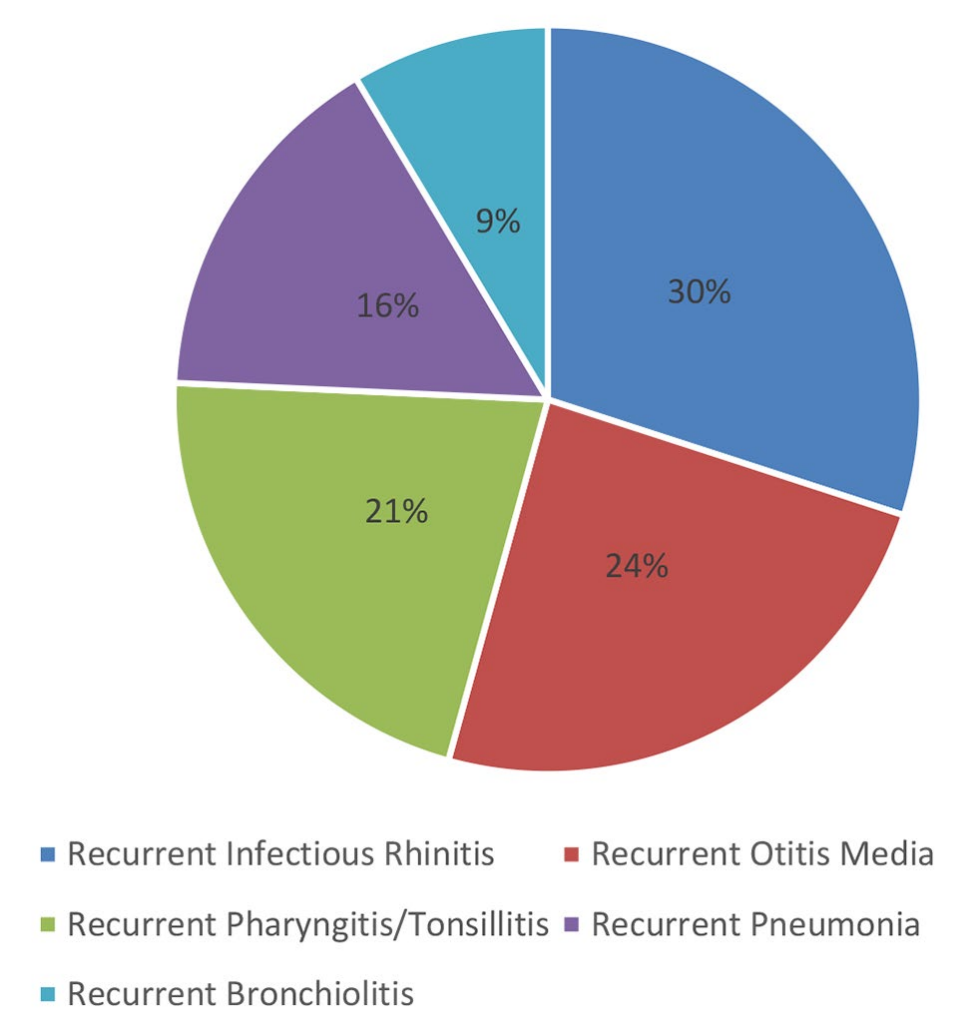
Frequency of the recurrent respiratory tract infections enrolled in the study

The most common RRTI was Infectious rhinitis which was diagnosed in 30% of the children, followed by otitis media (24%) and pharyngitis/tonsillitis (21%), Fig. 1. After giving probiotics for 2 weeks, we followed these patients for the next 6 months. There was a significant decrease in RRTIs at 6 months after the use of probiotics. The most significant decrease (p-value less than 0.05) was found in recurrent infectious rhinitis (17.14% percentage decrease) followed by recurrent otitis media and recurrent bronchiolitis. Table 3 shows the frequency and percentage of RRTIs before and after 6 months of probiotics, along with the percentage decrease in RRTIs.

**Table 1:** Demographics of the patients with recurrent respiratory tract infections (n = 70)

Demographics		Frequency	Percentage
Gender	Male	39	55.71
	Female	31	44.29
Age	6 months 12 months	29	41.43
	1 to 5 Years	23	32.86
	6 to 12 Years	18	25.71

**Table 2:** Presenting complaints of the patients presenting with recurrent respiratory tract infection

Presenting Complaints	Frequency	Percentage
Fever	51	72.86
Cough	48	68.57
Earache/ear discharge	19	27.14
Respiratory distress	9	12.86
Sore throat	18	25.71
Wheezes	32	45.71
Nasal discharge/sneezing/flue	23	32.86
Headache	6	8.57

**Table 3:** Comparison of recurrent respiratory tract infections before and after using the probiotics (n = 70)

Diagnosis	Before probiotics use		After Probiotics use		Percentage decrease in RRTIs	P-value
	Frequency	Percentage	Frequency	Percentage		
Recurrent Infectious rhinitis	21	30	9	12.86	17.14	0.02*
Recurrent Otitis media	17	24.29	7	10	10	0.03*
Recurrent pharyngitis/tonsillitis	15	21.43	6	8.57	12.85	0.07
Recurrent pneumonia	11	15.71	4	5.71	10	0.09
Recurrent bronchiolitis	6	8.57	3	4.29	4.28	0.04*

Asterisk (*) denotes the significant association

Chi Square test of association was applied

## Discussion

In recent years, the effect of probiotics on immune function has been a hot topic in many diseases. Studies have shown that microbiome given in the form of probiotics (whether pharmaceutical or natural foods) causes immunomodulation that leads to decreased episodes of gut and respiratory tract infections [17, 18]. In the gut, probiotics play a safeguarding role by improving intestinal barrier functionaries along with immune-modulatory functioning by generating and modulating helper T cells [19]. However, their effectiveness in the prevention of respiratory tract infections is not much studied. Studies done in pediatric and geriatric patients show that when probiotics are given in combination, fewer incidences of respiratory tract infections were observed as compared to placebo [19, 20].

In this study, we observed that when patients with RRTIs were given probiotics containing a combination of stains for two weeks, there was a significant decrease in RRTIs over the next six months. All RRTIs episodes decreased over the next six months but the most significant decrease was found in recurrent infectious rhinitis, recurrent otitis media and recurrent bronchiolitis. A randomized controlled trial (RCT) based systemic review done by de Araujo et al. showed that all those children who were given probiotics and followed over 6–7 months showed a decrease in new disease episodes, decrease in disease symptoms and duration of disease episodes as compared to placebo-controlled patients. The drawback of this systemic review is that it includes all RCTs from developed countries [21]. Another study by Li et al. also showed that children who received multi-strain probiotics for two months had lesser episodes of RTIs and the need for antibiotics was also decreased as compared to the control group [22].

The duration for which probiotics should be given to assess their effects is not variably studied. Some authors have given probiotics for longer durations while some for just a few days. In our study, we gave probiotics for two weeks and assessed whether it is effective in decreasing the severity and frequency of RRTIs over the next six months. Skovbjerg et al. gave probiotics for ten days only and found a decrease in respiratory disease symptoms, while Rautava et al. gave probiotics till 11 to 12 months of life and found decreased episodes of acute otitis media in the first year of life [23, 24]. Similarly, Li et al. gave multi-strain probiotics for two months and found that these children had lesser episodes of RTIs [22].

In our study, we gave a combination of probiotics that is lactobacillus and bifidobacterium and found that these are effective enough to decrease the episodes and severity of many of the RRTIs. Taipale et al. gave Bifidobacterium subspecies lactis in infants and found that it decreased the episodes of RTIs in infants [25]. Rutava et al. found that giving lactobacillus and bifidobacterium-containing probiotics to the infants before first two months of life, decreases the incidence of RRTIs and the need for antibiotics usage over the next 6–7 months of life [24]. A study done by Hojsak et al. on hospitalized Croatian children found that those who received lactobacillus probiotic-containing milk were less likely to suffer from RTI than the placebo group [26].

### Adherence and adverse events monitoring

Parents were given clear written instructions on how and when to give probiotics. Patients were advised follow-up at the time of completion of the probiotic course. Patients were assessed for adherence and any side effects of probiotics. No Adverse effects observed either during two weeks of probiotics use or soon after.

### Strengths and limitations of the study

As per the literature search, this is the only study on the topic from the region. This study includes almost all of the RTIs, unlike previous studies done on the role of probiotics in respiratory tract infections that were done on the selected disease like isolated otitis media, pneumonia or rhinitis etcetera. Limitations of this study are the small sample size and single study center. The present study did not consider malnutrition, poor vaccination coverage, poor sanitation, large joint families and other issues that affect the recurrent respiratory tract infections. The possible potential bias in this study was selection bias and statistical bias. However, we made every possible effort to minimize any bias affect our results.

## Conclusion

The results of our study indicate that the administration of probiotics may be useful in reducing recurrent respiratory tract infections among children. This six months trial has demonstrated that there was a significant decline in respiratory symptoms among study participants. The study also observed a significant decrease in respiratory diseases during the follow-up. Further large-scale, well-designed, randomized, multicenter trials are required to validate the current findings and determine the effectiveness of different strains of probiotics.

### Recommendations

We recommend the use of multi-strain probiotics in children, especially below 5 years with recurrent respiratory tract infections.

## Data Availability

Most of the data generated or analyzed during this study are included in this article. Limited data can be provided in person on request to the main author (Dr Imran Khan Laghari) at dr.imranlaghari@yahoo.com.
